# Surveillance for potentially zoonotic viruses in rodent and bat populations and behavioral risk in an agricultural settlement in Ghana

**DOI:** 10.1186/s42522-022-00061-2

**Published:** 2022-03-08

**Authors:** Richard Suu-Ire, Evangeline Obodai, Samuel Otis Bel-Nono, William Kwabena Ampofo, Jonna A. K. Mazet, Tracey Goldstein, Christine Kreuder Johnson, Brett Smith, Linda Boaatema, Theodore Worlanyo Asigbee, Joseph Awuni, Eric Opoku, PREDICT Consortium, Terra R. Kelly

**Affiliations:** 1 School of Veterinary Medicine, University of Ghana, Legon, Accra, Ghana; 2 Noguchi Memorial Institute for Medical Research, University of Ghana, Legon, Accra, Ghana; 3 One Health Institute, University of California, Davis, 1089 Veterinary Medicine Drive, Davis, CA USA; 4 Military Veterinarian (Rtd), P.O. Box CT2585, Accra, Ghana; 5 Zoological Pathology Program, c/o Chicago Zoological Society, 3300 Golf Rd., Brookfield, IL 60513 USA; 6 Accra Veterinary Laboratory, Veterinary Services Directorate, Ring Road East, Accra, Ghana; 7 Ghana Health Service, 28th February Road, Accra, Ghana

**Keywords:** Bats, Coronavirus, Ghana, Paramyxovirus, Zoonoses

## Abstract

**Background:**

In Ghana, the conversion of land to agriculture, especially across the vegetative belt has resulted in fragmented forest landscapes with increased interactions among humans, domestic animals, and wildlife.

**Methods:**

We investigated viruses in bats and rodents, key reservoir hosts for zoonotic viral pathogens, in a small agricultural community in the vegetation belt of Ghana. We also administered questionnaires among the local community members to learn more about people’s awareness and perceptions of zoonotic disease risks and the environmental factors and types of activities in which they engage that might influence pathogen transmission from wildlife.

**Results:**

Our study detected the RNA from paramyxoviruses and coronaviruses in rodents and bats, including sequences from novel viruses with unknown zoonotic potential. Samples collected from *Epomophorus gambianus* bats were significantly more likely to be positive for coronavirus RNA during the rainy season, when higher numbers of young susceptible individuals are present in the population. Almost all community members who responded to the questionnaire reported contact with wildlife, especially bats, rodents, and non-human primates in and around their homes and in the agricultural fields. Over half of the respondents were not aware or did not perceive any zoonotic disease risks associated with close contact with animals, such as harvesting and processing animals for food. To address gaps in awareness and mitigation strategies for pathogen transmission risks, we organized community education campaigns using risk reduction and outreach tools focused around living safely with bats and rodents.

**Conclusions:**

These findings expand our knowledge of the viruses circulating in bats and rodents in Ghana and of the beliefs, perceptions, and practices that put community members at risk of zoonotic virus spillover through direct and indirect contact with bats and rodents. This study also highlights the importance of community engagement in research and interventions focused on mitigating risk and living safely with wildlife.

## Introduction

Emerging zoonoses, the majority of which originate from human-wildlife interactions [1], can have devastating public health and socioeconomic impacts, as evidenced by the SARS-CoV-2 pandemic [2, 3]. While emerging zoonotic diseases are a major concern around the world, their impacts in low and middle-income countries are disproportionately high [4, 5]. This may be the result of a complex interplay of factors such as rapid human population growth, limited infrastructure and health workforce capacity, compromised immunity associated with comorbidities such as HIV/AIDS and/or parasitic diseases, and a greater dependence on animals and agriculture for livelihoods [4, 6].

Over the past few decades, our recognition of the role that wild animals play as hosts and/or reservoirs for emerging pathogens highlights the importance of understanding the human behaviors and other factors that bring people into direct and indirect contact with wildlife. Human activities along with the socio-economic, environmental, and ecological conditions that drive pathogen spillover from wildlife into human populations [6–8] are complex and the subject of ongoing intensive study [6]. Wildlife hosts play a critical role in disease dynamics, as they can serve as reservoirs and ongoing sources of infection for domestic animals and humans, facilitate movement of some pathogens via migration and trade, and provide opportunities for pathogen evolution through host switching and genetic exchange [9, 10].

Certain species within the orders Chiroptera and Rodentia are increasingly recognized as important evolutionary hosts of emerging viral zoonoses. In many rural and urban areas across Africa, large bat roosts can be found near human activity. Bats are increasingly adapting to peri-urban and urban environments. For example, more than one million fruit bats roost within the limits of Accra, the capital city of Ghana, where hunting and sales of bats are important economic activities [11–13]. Bats are utilized for food and are commonplace in the bushmeat value chain in Ghana [12, 14]. Bats also provide critical ecosystem services such as seed dispersal, pollination, and control of insect pests [15]. Some bat species are known or suspected to be evolutionary hosts of high consequence zoonotic pathogens, including filoviruses (Ebola and Marburg viruses), Hendra and Nipah viruses, rabies virus, and severe acute respiratory syndrome coronaviruses [6, 16, 17]. Rodents are also known to harbor a plethora of zoonotic pathogens of public health importance [18, 19], including hantaviruses causing pulmonary syndrome and hemorrhagic fever with renal syndrome [20] and arenaviruses causing lymphocytic choriomeningitis; Lassa fever; and Argentine, Bolivian, Venezuelan, and Brazilian hemorrhagic fevers [21]. Similar to bats, rodents are diverse [22] and well-adapted to a wide range of habitats [23], including peri-urban environments, where they benefit from anthropogenic activities, including agriculture.

Transmission of zoonotic viruses from wildlife to people is thought to occur across a range of human-wildlife interfaces, with spillover commonly associated with human-wildlife contact in peridomestic and agricultural settings [7]. Anthropogenic changes to the landscape can dramatically alter the types and availability of resources, especially food sources, to wild animals [24]. Some wildlife species adapt well to human-dominated landscapes taking advantage of these artificial food sources [25, 26]. For example, commensal rodents, attracted by easy access to food, have been implicated in spillover and transmission of zoonotic pathogens to humans in and around homes and agricultural fields where food and nesting sites are plentiful [7, 27]. Similarly, in the tropics, bats move according to shifts in availability of food resources and habitat [11, 28]. Depending on home range size, some species, such as *Eidolon helvum*, can travel > 90 km from day roosts to foraging sites in a single evening [29, 30].

Rural communities in Ghana are potentially vulnerable to pathogen spillover at the peridomestic and agricultural interfaces. Approximately 46% of the Ghanaian population is engaged in agriculture [31], and crop production along with human settlement have been identified as major drivers of deforestation and land use change in Ghana [32, 33]. The migration to rural agriculturally rich areas in Ghana has intensified in recent decades largely in response to worsening poverty [34]. Along with migrant farmers, comes increased anthropogenic pressures to support livelihoods, such as land clearing, mixed farming, and hunting [33, 34]. With this encroachment, there is increased contact with people and domestic animals, as wild animals migrate out of degraded environments into areas with human activity. As in many countries, livestock in rural Ghana live in close contact with people [35], and architectural structures permit easy entrance to human dwellings by both domestic and wild animals seeking food and shelter [16, 36]. In these settings, there is a critical need to support livelihood practices while identifying ways in which local community members can decrease their risk for exposure to potentially zoonotic pathogens.

The United States Agency for International Development’s (USAID) Emerging Pandemic Threats (EPT) PREDICT-2 project is an example of a project that strengthened One Health capacities for early detection, rapid response, and development of risk reduction strategies for zoonotic viruses of pandemic potential [37]. One Health is based on a systems approach, which includes multiple disciplines working together at the local, national, and global levels, to attain optimal health for people, animals, and our environment [38]. The PREDICT project’s virus surveillance efforts were targeted at high-risk human-animal pathogen transmission interfaces at sites with environmental, ecological, and socioeconomic conditions hypothesized as drivers of disease emergence and spread. As part of the PREDICT project, this study aimed to engage small-scale agriculturalists living amongst forest fragments in the rural vegetative belt of Ghana to understand the human-wildlife interactions that occur in their communities, test for the presence of viruses in local bat and rodent populations, determine the potential pathways for contact with these high-risk wildlife taxa, and conduct campaigns to enhance awareness of the potential for exposure to zoonotic viruses and strategies to mitigate risk.

## Material and methods

### Study site and population

**Fig. 1:**
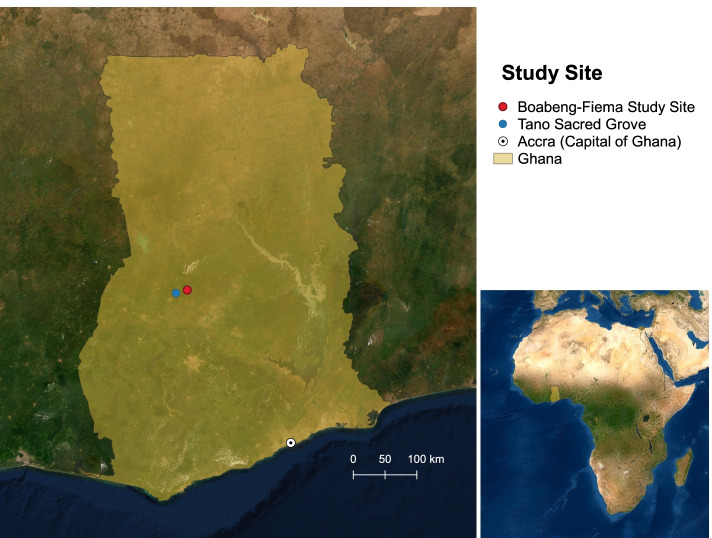
Map of Ghana showing the study site at the villages of Boabeng and Fiema where bats and rodents were sampled from February 2017 – December 2018 and the nearby Tano Sacred Grove, a protected area that provides habitat for large colonies of fruit bats in Ghana. The bats and rodents were tested for five families of viruses with pandemic potential (coronaviruses, paramyxoviruses, flaviviruses, influenza viruses, and filoviruses). The villages of Boabeng and Fiema are located along the vegetative belt in the Bono East Region of Ghana

Data for this manuscript, which include viruses detected in bats and rodents and behavioral information on human-wildlife contact were collected from February 2017 to December 2018 in two adjacent villages in the Bono East Region in Ghana (7^o^ 43′N, 1^o^42’W; Fig. 1). The villages, which have a combined population of 3754 people [39], were chosen as a site for this study as this region has undergone recent and on-going anthropogenically-induced landscape change characterized by deforestation with a patchwork of protected mixed deciduous forest fragments interspersed among villages, orchards, and agricultural fields [33]. The protected forest is a managed sanctuary providing habitat for two revered non-human primate species (black and white colobus (*Colobus vellerosus*) and Lowe’s mona monkey (*Cercopithecus campbelli*)).

Communities at the site are primarily small-scale smallholder farmers who rely on crop and livestock production and to a lesser degree hunting for their livelihoods. The site is within the forest-savannah transition zone along the vegetative belt of Ghana and is characterized by a moderate climate and fertile soils in which a range of subsistence and cash crops are produced. Cashew nut (*Anacardium occidentale*), mango (*Mangifera* sp.), fig (*Ficus* sp.), and neem (*Azadirachta indica*) trees provide roosting and foraging habitat for a diversity of fruit bats that are resident or migrating through the area. The Tano sacred grove, a protected area 20 km north of the study site, hosts diverse bat species and the largest colony of straw-colored fruit bats (*Eidolon helvum*) in Ghana, with an estimated 2 million bats [11, 40]. Bats from this grove travel to the surrounding orchards to feed. In addition, several species of rodents make their homes in and around human dwellings and in the agricultural fields at the study site, bringing these wild animals into close contact with community members and their livestock. Farmers in these communities primarily raise local breeds of sheep, pigs, domestic fowl (chickens and ducks), and cattle. We collected data on characteristics of the site to supplement observational data, including habitat types, human population density, species and population estimates of domestic and wild animals present, water sources, anthropogenic changes, and types of human-animal contact.

### Wildlife sample collection

Bats and rodents were humanely sampled (and released back to the wild) from February 2017 to December 2018. Sampling occurred during both the rainy and dry seasons targeting a minimum of 100 animals per taxa per season each year. Seasons were classified using precipitation data from the Ghana National Climate Change Committee with the dry season ranging from November to March (mean annual rainfall of 215 mm) and the rainy season from April to October (mean annual rainfall of 1250 mm) [41].

#### Bat and rodent sampling

Bats were captured using 6–18 m mist nets. The mist nets were set before dusk and opened for trapping three to four hours before dawn when bats returned to their roosts from foraging. The mist nets were actively monitored throughout the trapping session, and bats were extracted from the net as soon as possible after capture [42] by personnel wearing full personal protective equipment (PPE) for biosafety. Each bat was placed into a porous cotton bag and kept in a cool location until sampling. Rodents were live-captured using Sherman live traps (22.9 × 8.9 × 7.6 cm^3^) and locally produced wire mesh traps, baited with fish. Rodent traps were set at night in the agricultural fields and with the resident’s permission, around and within houses and outbuildings. Traps were collected in the early morning and placed in a cool, shady location during processing. Rodents were anesthetized with isoflurane using the open drop method [43] for sampling. Rodents and bats were weighed, and morphometric measurements were obtained using calipers, including body length (tip of nose to base of tail), tail length, ear length, and hind foot length for rodents and forearm length for bats. Data on health status, age class, sex, and reproductive status (pregnant, lactating) were also collected.

Oral, urogenital, and fecal swabs were collected in duplicate using sterile, polyester-tipped swabs and placed in viral transport medium (VTM) and TriReagent (Trizol) and stored in liquid nitrogen until transferring to an ultra-low freezer (− 80 °C). Blood samples were collected from the lateral tail or saphenous vein in rodents and from the brachial or cephalic veins in bats. An aliquot of blood was preserved in VTM and stored in liquid nitrogen until transferring to an ultra-low temperature freezer (− 80 °C). Serum was also archived from animals for which the blood volume was sufficient (blood samples were not taken in excess 1% of the total body weight). All bats and rodents were temporarily marked with non-toxic nail polish or markers applied to the claws or fur to avoid repeated sampling within the same season’s capture event. The animals were all apparently healthy and released following sampling.

#### Species identification

Species identification was confirmed by DNA bar coding of the cytochrome b (Cytb) and cytochrome oxidase subunit 1 (CO1) mitochondrial genes [44] for PCR-positive individuals. The PCR amplicons were sequenced and BLASTed against reference sequences in GenBank. Sequences with > 97% sequence identity were classified to the host species. Sequences that did not meet this threshold were classified to the genus level. DNA barcoding was also performed on a subset of the PCR-negative samples.

### Community engagement and questionnaire administration

We administered questionnaires among the local community members to learn about people’s awareness and perceptions of zoonotic disease risks and the environmental factors and types of activities in which they engage in that might influence the risk of pathogen transmission from wildlife to humans (human demographics, livelihood activities, types of animal contact, and food safety and sanitation practices).

Prior to initiating the study, the project team met with local officials and community leaders to discuss the goals of the project. With permissions from the local authorities, our team conducted household visits and made announcements in the villages to inform the community members of the study. All messages were communicated in the local dialect using lay language to convey the study purpose, eligibility, potential risks and benefits of participation, and the time during which the study would take place. The team selected all households in the village for participation. Only one person per household was recruited and efforts were made to include participants across a range of age and gender.

The aims of the study were communicated in the local language, and written informed consent was obtained from all study participants. Questionnaires were administered to collect demographic and livelihood information, travel history, and data on interactions with domestic and wild animals. The questionnaires were written in English and translated into the local language (Twi) during administration.

### Virus detection and discovery

Testing of the oral and rectal swab samples was performed at the UC Davis One Health Institute Laboratory in Davis, California and Veterinary Services Directorate, Ministry of Food and Agriculture in Ghana. A 250 μl aliquot of each sample was utilized for RNA extraction. RNA was extracted using Direct-Zol RNA columns (Zymo Research Corp), and 8 μl RNA was reverse transcribed into cDNA transcription using Superscript III (Invitrogen Corp, Carlsbad, CA).

The housekeeping gene, b-actin, was targeted as an internal control for the presence of amplifiable nucleic acid in the RNA extracts [45]. The RNA extracts were then screened via consensus PCR targeting conserved RNA regions for corona- [46, 47], paramyxo- [48], flavi- [49], influenza [50], and filo- [51] viruses. Bands of the expected size for each assay were excised and purified using the Qiaquick kit (Qiagen Inc.). Purified PCR products were cloned (pCR4-TOPO vector; Invitrogen Corp.) and sequenced (ABI 3730 Capillary Electrophoresis Genetic Analyzer; Applied Biosystems, Inc., Foster City, CA). Sequences were analyzed and edited using Geneious Prime (Version 2019.1.3), uploaded into Genbank, and compared with known sequences. Sequences were classified as belonging to viral taxa according to established cut-offs and methods [52]. Virus sequences sharing ≥90% identity to another sequence in the GenBank database were classified as a known virus sequence, while viral sequences sharing less than 90% identity to a known sequence were considered novel viruses and named sequentially with other previously unreported virus sequences detected as part of the PREDICT project. Virus isolation was not attempted for any of the positive samples.

### Data analysis

Statistical analyses of the survey and virus detection data were performed using R v3.6.0 [53]. Responses to the survey were coded, and descriptive statistics were calculated. The frequencies of responses related to hunting and slaughtering of animals were evaluated for differences by gender and age of the respondents using Chi-square tests. Given the high frequency of Kenya bat coronavirus/BtKY56/ detections in *Epomophorus gambianus* bats, we conducted analyses to explore associations between host demographics, season, and coronavirus RNA positive samples. The Fisher’s exact test was used to compare the proportion of positive Kenya bat coronavirus/BtKY56/BtKY55 results in *E. gambianus* bats across sex, age class, season, and specimen type. Logistic regression models were then constructed to explore the relationships between these factors and a positive Kenya bat coronavirus/BtKY56/BtKY55 RNA result in the *E. gambianus* bats.

## Results

### Virus detection

A total of 418 bats and 293 rodents were sampled over eight sampling events between February 2017 and December 2018. *Epomophorus gambianus* was the most common bat species captured, making up 86% (*n* = 341) of the sample. Other bat species commonly captured were *Eidolon helvum* (*n* = 9)*, Epomops buettikoferi* (*n* = 3)*, Epomops franqueti* (*n* = 18)*,* and *Mops condylurus* (*n* = 25)*.*

Of the 418 bats tested, 17% (71/418) of the bats were positive for viral RNA from one or more viruses. Five unique viruses were detected in the bats, including one new betacoronavirus (PREDICT CoV-102, Genbank Accession Number MT082204), one new paramyxovirus (PREDICT PMV-15, Genbank Accession Number MT125230), and three previously reported coronaviruses (Table 1). None of the samples tested positive for influenza-, flavi-, or filovirus RNA. A co-infection with Chaerephon bat coronavirus/Kenya/KY22/2006 and Kenya bat coronavirus/BtKY56/BtKY55 was detected in one adult *Mops condylurus* bat.

**Table 1:** Number and percentage of bats testing positive for viral RNA in the Boabeng-Fiema area in Ghana, from February 2017 – December 2018. Results are presented by bat species with RNA positive samples, season (dry/rainy), and specimen type (oral/rectal swabs). Bats were tested for five viral families with pandemic potential (coronaviruses, paramyxoviruses, flaviviruses, influenza viruses, and filoviruses)

Taxonomic Family	Species Name	Total Number Sampled	Virus RNA Detected	Percentage of Positive Bats (positive/total)				
				No Positive	Rainy Season	Dry Season	Oral Swabs	Rectal Swabs
*Pteropodidae*	*Epomophorus gambianus*	341	*Kenya bat coronavirus/BtKY56/BtKY55*	61	39% (39/100)	9.1% (22/241)	27	34
			*Eidolon bat coronavirus*	1	0% (0/100)	0.4% (1/241)	0	1
			*PREDICT_CoV-102*	1	0% (0/100)	0.4% (1/241)	0	1
	*Eidolon helvum*	9	*Eidolon bat coronavirus*	1	0% (0/0)	11.1% (1/9)	0	1
	*Epomops buettikoferi*	3	*Kenya bat coronavirus/BtKY56/BtKY55*	1	0% (0/0)	33.3% (1/3)	0	1
	*Epomops franqueti*	18	*Kenya bat coronavirus/BtKY56/BtKY55*	1	20% (1/5)	0% (0/13)	0	1
*Molossidae*	*Mops condylurus*	25	*Chaerephon bat coronavirus/Kenya/KY22/2006*	1	0% (0/0)	4.0% (1/25)	1	0
			*Kenya bat coronavirus/BtKY56/BtKY55*	4	0% (0/0)	16.0% (4/25)	4	0
			*PREDICT_PMV-15*	1	0% (0/0)	4.0% (1/25)	1	0

The analyses to explore associations between host demographics, season, specimen type and Kenya bat coronavirus/BtKY56/BtKY55 shedding in *Epomophorus gambianus* bats revealed that a higher proportion of bats sampled during the rainy season (37% (37/100) were positive for coronaviruses than bats sampled during the dry season (9% (23/241); Table 2). In fact, *E. gambianus* bats were over five times more likely to test positive for coronavirus RNA during the rainy season as compared to the dry season (OR = 5.6, 95% CI: 3.1–10.1) (Table 3). In this analysis, detection of coronavirus RNA did not differ by age class or sex of the bat. Kenya bat coronavirus/BtKY56/BtKY55 RNA was detected more frequently in rectal swabs (35/341) than oral swabs (25/341; however, this difference was not statistically significant. In five of the bats, coronavirus sequences were confirmed in both oral and rectal swabs from the same individual.

**Table 2:** Percentages of bats positive for coronavirus RNA by host demographics (sex and age class) and season (dry/rainy). *P*-values correspond to Chi-square tests evaluating the associations between coronavirus RNA positive bats and sex and age class of the bats as well as season

		% (No. Positive/Total Sampled)	*P*-value
Sex			0.02
	Female	13.1% (31/236)	
	Male	21.9% (40/182)	
Age Class			0.5
	Adult	16.1% (40/248)	
	Subadult	18.8% (32/170)	
Season			< 0.0001
	Dry	10.0% (31/310)	
	Wet	38.0% (41/108)

**Table 3:** Factors significantly associated with coronavirus RNA positive results in *E. gambianus* bats sampled from February 2017 – December 2018 in the Boabeng-Fiema area in Ghana as identified by logistic regression analyses

Predictor	Odds ratio (95% C.I.)	*P*-value
Season (Rainy)	5.6 (3.1–10.1)	< 0.0001

*Praomys derooi* was the most common rodent species captured at this site making up 98% of the rodents sampled (287/293). *Heimyscus fumosus* (*n* = 4), *Mastomys natalensis* (*n* = 1), and *Taterillus gracilis* (*n* = 1) were also captured and sampled for the study. Of the 293 rodents tested, 2% (6/293) of rodents were positive on virus screening. RNA from a new paramyxovirus was detected in six *Praomys derooi* rodents (PREDICT PMV-171, GenBank numbers: MT 063672; MT125231; MT125232; MT125233; MT125234; MT125235). The virus RNA was detected in rectal swabs from four rodents and oral swabs from two rodents. No positive viral findings were found in the influenza, corona-, flavi-, or filovirus groups.

### Contact with animals and community perceptions of zoonotic disease risks

Based on field observations, potential pathways for exposure to bats and rodents were primarily in people’s homes and through livelihood activities, such as farming and hunting. At the study site, fruit bats roosted in the nearby orchards and in residential areas, providing opportunities for exposure of farmers and community members to bat urine and feces and to fruits partially consumed by bats. Free-ranging livestock, including domestic fowl, pigs, and sheep, traverse through and forage in the patchwork of protected forest and agricultural fields that provide habitat and food resources for wildlife, providing opportunities for pathogen spillover and amplification in domestic animal hosts.

Our team administered questionnaires to 264 community members who consented to participate in the study. Among the participants, 48% were female and 51% were male. The median age of the respondents was 43 years (range: 16–85 years). The majority of community members had completed some level of education with 46% of respondents reporting completion of primary school, 33% secondary school, and 3% college/university level. Most participants (73%) were smallholder farmers engaging in crop and livestock production. Livelihood activities centered around farming, with the majority of participants (73%) engaging in food and cash crop production (yam, maize, and cashew) and livestock production (domestic fowl, sheep, and pigs). Animals were raised in extensive semi-scavenging systems where they free-range during the day and return to mixed animal outdoor enclosures at night. To a lesser extent, community members also engaged in hunting, timber harvest, construction, meat/food processing, teaching, and healthcare for their livelihoods.

Raiding of crops by wildlife was common in the communities, with almost all respondents (98%) reporting crop raiding by wild animals (primarily by rodents, bats, primates, and wild birds). Deterrents were frequently applied and included trapping, shooting, and poisoning the animals to mitigate loss of crops. Nearly all respondents reported animals entering their homes (99%), including primates (92%), rodents and shrews (29%), and bats (5%), in addition to domestic fowl (88%) and sheep (79%). Approximately three-quarters of respondents also reported consuming food that had been handled or damaged by animals, and 70% observed animal excreta in or near household food sources. Over half (52%) of the community members did not store their food in closed containers, facilitating access by domestic and wild animals.

Approximately, 28% of community members reported hunting wild animals at some time during their lifetime with 11% reporting hunting in the previous year. Rodent and bat hunting were observed at the site as a supplemental livelihood activity. Bushmeat sales were observed along the roadsides at the study site. Hunting and slaughtering of animals were primarily male activities (89% of hunters were male and 75% of respondents reporting slaughtering of animals during their lifetime were male), and the associations between gender and hunting and animal slaughtering were significant (both *p* < 0.0001). Over half of the respondents (54%) either did not know or did not perceive any disease risks associated with animal butchering, and 19% reported not taking any preventive measures or seeking treatment when injured while butchering. Further, 62% of individuals reported a disease outbreak in animals (domestic fowl and sheep) during the previous year, yet only 18% of these respondents indicated that sick animals were treated, quarantined, or culled. Some individuals also reported consuming animals (wild and domestic) they found ill (30%) or dead (13%).

To address gaps in awareness and mitigation strategies around the potential for pathogen transmission risks from wildlife species to people, the PREDICT team developed risk reduction and community outreach tools focused on living safely with bats and rodents [54]. Similar materials focused on risk reduction messages related to interactions with non-human primates were developed in collaboration with the Breakthrough Action project consortium. The resources, incorporating messages related to reducing zoonotic disease risk, were utilized in an outreach campaign conducted by wildlife and health promotion officers in the local community to discuss zoonotic disease risks and strategies that community members could take to minimize interactions with these species. The materials also included educational messages on the important ecological niche these species fill, and risk mitigation was focused on strategies that protect these species. The chiefs, heads of local government departments, community health workers, and public health and environment officers were engaged in developing the community outreach materials. Traditional rulers (chiefs), religious leaders, traditional healers, sanctuary staff, assemblymen, and opinion leaders were engaged with the goal of reaching additional individuals who communicate key messages from the campaign to community members. Educational flyers were provided for schools, ecotourism centers, and community clinics.

## Discussion

This study revealed that rural agriculturalist communities living amongst a fragmented landscape in rural Ghana have close and frequent contact with wildlife species. Fruit bats feed in orchards, roosting near human habitation, and are hunted and prepared for meat and for sale in this region. In addition, insectivorous bats roost within homes and sleeping quarters. Rodents are also found in people’s homes and agricultural fields allowing for contamination of crops, food stores, and water supplies. They are also harvested, consumed, and sold for bushmeat. Taken together, these observations highlight several potential pathways for zoonotic pathogen spillover from these wildlife taxa into humans, including direct (hunting and consumption) and indirect pathways (via contact with urine, feces, and saliva). These findings, in addition to the lack of awareness and perception of zoonotic disease risk associated with high-risk activities, illustrate the potential risk present at this rural agricultural interface if these animals carry zoonotic viruses.

Previous studies conducted in Ghana have also documented close interactions between humans, rodents, and bats, including hunting bats and rodents for bushmeat, residing among bat roosts in urban and rural settings, and visiting bat caves for tourism and religious practices [12, 55, 56]. Our study contributed additional insights into the types of contact occurring among wildlife, domestic animals, and people in agricultural communities living in Ghana and the potential for zoonotic disease transmission at this interface.

Although we did not detect known zoonotic viruses in the bats and rodents sampled in this study, we identified RNA sequences from several coronaviruses in bats as well as RNA from a novel paramyxovirus for which the zoonotic potential is unknown. Further characterization would be required to elucidate whether these viruses can jump species barriers and infect humans. Known zoonotic viruses within the viral families targeted for surveillance in this study are rare, so the lack of detection of these viruses in this study was not an unexpected finding. Bats have been shown to harbor a wide range of novel viruses belonging to a number of different virus families. Bats harbor the largest diversity of coronaviruses among mammals and two coronavirus genera, α- and β-CoVs, have been widely detected in bats across the world [52, 57, 58]. Bats are presumed hosts and reservoirs of important zoonotic coronaviruses including progenitors of Severe Acute Respiratory Syndrome coronavirus (SARS)-CoV (in *Rhinolophus bats*) and Middle Eastern respiratory syndrome coronavirus (MERS-CoV) (in Egyptian tomb bats, *Taphozous perforatus*) [59–61]. The emergence of these important human pathogens highlights the importance of further investigation of coronaviruses circulating among bat populations.

The frequency of coronavirus RNA positive bats and detection of different coronaviruses among the same species in this study is consistent with previous studies conducted across Africa [62–64]. These findings, in combination with the detection of a co-infection with one alphacoronavirus and one betacoronavirus in one of the bats, lends further support to the body of evidence that bats serve as hosts of a high diversity of coronaviruses, that certain coronaviruses are found in more than one bat host, and that bats can serve as hosts for multiple coronaviruses [65–69]. Collectively, these findings are important in that viruses with a higher host plasticity are associated with higher risk of spillover into human populations [7] and that cross-species transmission increases the probability of recombination and emergence of new coronavirus strains [70, 71].

We found that bat samples collected during the rainy season were more likely to be positive for Kenya bat coronavirus/BtKY56/BtKY55 than samples collected during the dry season. Although age class was not significantly associated with coronavirus shedding in *E. gambianus* bats in this study, the seasonality of shedding with higher detection during the rainy season is likely due to weaning and introduction of new pups resulting in higher numbers of susceptible individuals in the population as has been reported in other species of bats elsewhere [52, 72, 73]. For example, Montecino-Latorre et al. (2020) reported that coronavirus shedding among several species of bats in East Africa was higher during the weaning period irrespective of the age of the bat [72]. In addition, previous studies have shown that coronavirus transmission may be favored by high colony density and birth pulses in *Myotis macropus, Myotis myotis,* and *Eidolon helvum* bats [72, 74, 75].

Our study is the first to report a paramyxovirus detected in an insectivorous bat in Ghana. Fruit bats, also known as ‘flying foxes’, of the genus *Pteropus* are the reservoir for Nipah virus and Hendra virus, viruses within the *Paramyxoviridae* family that pose important zoonotic threats to people and domestic animals. In Ghana, Achimota virus 1 (AchPV1) and Achimota virus 2 (AchPV2), novel rubulaviruses in the *Paramyxoviridae* family, have been detected in *E. helvum* fruit bats [76]. Serological evidence of human infection with AchPV2 suggests potential spillover of the viruses from bats to human populations or cross-reaction with other paramyxoviruses [77]. No cases of human illness associated with the viruses have been documented. However, animal infection studies of AchPV1 and AchPV2 in laboratory animals (ferret, guinea pigs, mice) reported seroconversion, immunohistological evidence of infection, and viral shedding in the ferrets and guinea pigs indicating that Achimota viruses can cross the species barrier [78]. Recent studies have also described the detection of paramyxoviruses in insectivorous bats in Europe [79] and the south-west Indian Ocean [80] and in fruit bats in China [81], Indonesia [82], Australia [83], and Africa [76, 84, 85]. Further research is needed to improve our understanding of paramyxoviruses circulating among diverse bat populations, and characterization is needed to understand if they pose potential public health risks.

Novel paramyoxviruses were detected in six Deroo’s mice (*Praomys deroo* in the family *Muridae*) in this study. This, to our knowledge, represents the first report of paramyxoviruses in rodents in Ghana. The Deroo’s mouse is a common species found living in and around human dwellings in the savanna and urban areas in Ghana, Togo, and Benin [86]. The majority of the rodents captured for this study were trapped inside people’s homes demonstrating the opportunities for human-rodent contact and subsequent potential risk to human health [87]. New paramyxoviruses have also been detected in rodents in recent studies in Kenya [88], South Africa, Zambia, Australia, and Asia [82]. Lee and colleagues [89] identified two new paramyxoviruses in *Apodemus agrarius* rodents which were found to infect human cell cultures highlighting the importance of additional studies to further characterize rodent-borne paramyxoviruses.

To address gaps in awareness and mitigation strategies around contact between wildlife species and people, we partnered with the community at the study site to develop educational materials and outreach tools (flyers, brochures, and community signage) and to conduct outreach campaigns focused on reducing contact with animals while conserving wildlife and their important ecosystem services. Emerging zoonoses and outbreaks in combination with rapidly deployed interventions can have devastating impacts on the livelihoods of communities. It is therefore critical to focus on community engagement and co-development of mitigation strategies in order to strike a balance between reducing risk, ensuring people’s livelihoods, and protecting wildlife and the ecosystem services these species provide.

## Conclusion

Our study detected the presence of RNA from known and novel coronaviruses in bats and from novel paramyxoviruses in bats and rodents in peri-domestic and agricultural settings in Ghana. The zoonotic potential and public health risk of these viruses are currently unknown and require further study. The results from this study suggest there is high interaction between humans and wildlife in the area, which is largely driven by livelihood activities. Exposure risk is further complicated by a lack of awareness of zoonoses among community members and the perception that contact with bats and rodents has minimal risk. Further work is needed to better understand the ecology of these viruses, including their zoonotic potential, the human-bat and human-rodent interactions that are associated with greater zoonotic disease risk, and the best strategies for communities to mitigate the risk of spillover that take into consideration the importance of wildlife conservation as well as local livelihoods.

## Data Availability

The datasets generated during and/or analyzed during the current study are publicly available in the USAID Data Development Library (DDL). Unrestricted data can be accessed by searching for PREDICT from the DDL home page: https://data.usaid.gov/. Restricted data, primarily from human interviews, requests should go through the USAID formal request process at: https://data.usaid.gov/access-requeste. The datasets during and/or analyzed during the current study would be available from the corresponding author on reasonable request. All data generated or analyzed during this study are included in this published article.
